# Identification and study of a *FBN1* gene mutation in a Chinese family with ectopia lentis

**Published:** 2012-02-24

**Authors:** Hongyi Li, Wei Qu, Bo Meng, Shuihua Zhang, Tao Yang, Shangzhi Huang, Huiping Yuan

**Affiliations:** 1 Department of Ophthalmology, the 2nd Affiliated Hospital of Harbin Medical University, Department of Ophthalmology Key laboratory, Harbin Medical University, Harbin, China; 2 Department of Medical Genetics, Institute of Basic Medical Sciences, Chinese Academy of Medical Sciences & Peking Union Medical College, WHO Collaborating Centre for Community Control of Hereditary Diseases, Beijing, China

## Abstract

**Purpose:**

To identify the mutation in the fibrillin-1 gene (*FBN1*) in a Chinese family with ectopia lentis (EL) and to predict the structural and functional consequences of the mutation.

**Methods:**

Patients and family members were given complete physical, ophthalmic, and cardiovascular examinations. Genomic DNA was extracted from leukocytes of venous blood of three affected and three unaffected individuals in the family, and 100 healthy controls. All 65 coding exons and their flanking intronic boundaries of *FBN1* were amplified in the proband by polymerase chain reaction, followed by direct sequencing. The mutation identified in the proband was screened for in other family members and 100 healthy controls by direct sequencing. Protein conservation analysis was performed in seven species using an online ClustalW tool. Protein structure was modeled based on the Protein data bank and mutated in PyMOL 1.1r1 to predict the structural and functional consequences of the mutation.

**Results:**

A heterozygous c.2262A>G change in exon 18 of *FBN1* was detected in the proband, which resulted in the substitution of tyrosine by cysteine at codon 754 (p.Y754C). This mutation was also present in the affected family members, but absent in other unaffected family members and 100 healthy controls. The mutant residue, located in the calcium binding epidermal growth factor-like7 domain, was highly conserved among mammalian species. The mutation could probably affect the disulfide bond formation of the domain and calcium binding of the adjacent domain, which would induce a critical functional change of the domain itself and neighboring domains.

**Conclusions:**

We indentified a p.Y754C mutation in FBN1, which is the causative mutation for EL in this family. This missense mutation introduced an additional cysteine residue by substitution of a highly conserved tyrosine residue within the cbEGF-like7 module.

## Introduction

Ectopia lentis (EL; OMIM 129600) is an inherited connective disorder characterized by lens dislocation connected with stretched or discontinuous zonular filaments [1]. It often occurs as one of the symptoms of Marfan syndrome (MFS; OMIM 154700), an autosomal dominant disorder that is characterized by manifestations mainly involving the cardiovascular, skeletal, and ocular systems [2]. The diagnosis of MFS is made according to the Ghent nosology [3]. Isolated EL or predominant EL with relatively mild skeletal features belongs to Marfan-related disorders as it does not satisfy the Ghent criteria.

Mutations in the gene of human fibrillin-1 (*FBN1*), fibrillin-2 (*FBN2*), transforming growth factor-β receptor-1 (*TGFBR-1*), and transforming growth factor-β receptor-2 (*TGFBR-2*) cause MFS [4-7]. Isolated or predominant EL is mainly caused by mutations in *FBN1* on chromosome 15q21.1 [4]. Human FBN1, a 350 kDa modular glycoprotein, is a major component of the 10–12 nm extra-cellular matrix (ECM) microfibrils [8]. The structure of fibrillin-1 reveals a highly repetitive protein that contains three repeated modules: 47 epidermal growth factor (EGF)-like modules (43 calcium binding (or cb) EGF-like modules and 4 non-cb EGF-like modules), seven transforming growth factor-binding (or TB) protein like modules (8 Cys/TB), and two hybrid modules [9]. To date, over 600 *FBN1* mutations spread over the entire gene have been registered in the Universal Mutation Database (UMD)-FBN1 database for MFS and its related diseases [10]. Missense mutations account for a major proportion (more than 60%) of the pathogenic mutations, and the majority of these mutations are localized in cbEGF domains (including those which result in substitution of calcium-binding residues or cysteine residues involved in the formation of disulphide bonds) [11,12].

Presently, no genotype/phenotype correlations have been identified except for neonatal MFS [13]. Besides, some recent studies showed strong correlations between isolated or predominant EL and cysteine substitutions, regardless of its location within the protein [14]. As a result, long-term study on genotype/phenotype correlations for MFS and its related diseases is vital, and molecular analysis of *FBN1* is becoming important for the possibility of prenatal diagnosis and detecting at-risk individuals at an early stage for awaking of serious output of the disease.

In the present study, we investigated a two-generation family affected with EL and found a missense mutation in the cbEGF-like7 domain of FBN1. The mutation found in affected individuals was not observed in any of the healthy ones. We predicted here the structural and functional consequences of the mutation and demonstrated the crucial role for tyrosine at this position in cbEGF-like domains.

## Methods

### Subjects

**Figure 1:**
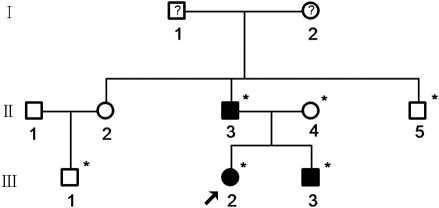
The pedigree of the family. Squares and circles indicate males and females, respectively, and the darkened symbols represent the affected members. Symbols with a question mark in the center indicate that the member is not diagnosed clearly. The asterisks indicate the subjects participating in this study. The patient above the arrow is the proband.

The family history revealed three affected members with EL. All the patients (II:3, III:2, and III:3) and three non-carrier members, including a spouse (II:5, III:1, and II:4), of the family were given complete physical, ophthalmic, and cardiovascular examinations after obtaining informed consent (Figure 1). One hundred control subjects without features of EL or MFS were also recruited. The study was approved by Harbin Medical University Ethics Committee (Harbin, China).

### Genomic DNA preparation

Whole blood from six available members of the family (II:3, II:4, II:5, III:1, III:2, and III:3) and one hundred unrelated controls were collected in tubes containing EDTA as an anticoagulant. Genomic DNA was extracted using the TIANamp Blood DNA Kit (Tiangen Biltech Co. Ltd, Beijing, China) according to the manufacturer’s protocol.

### Mutation analysis of *FBN1*

All 65 coding exons and flanking intronic regions including splice sites of *FBN1* were amplified by polymerase chain reaction (PCR) using a set of primers listed in Appendix 1. The PCR products were subsequently purified with a TIANgel Midi Purification Kit (Tiangen Biltech Co. Ltd) and sequenced with an ABI 3130XL Genetic Analyzer (Applied Biosystems, Foster City, CA). Sequencing results were assembled and analyzed using the Chromas 2.22 software (Technelysium Pty. Ltd., QLD, Australia) with reference sequence (NG_008805) on the NCBI website. The mutation was confirmed by bidirectional sequencing.

### Protein structure analysis

Orthologs of *FBN1* were identified with the UMD and NCBI websites, and sequences were aligned using an online ClustalW tool. Schematic of the cbEGF-like domain of human fibrillin-1 was used to assess the possible impact of the mutation at the secondary structure level [15]. A homology 3D model of the cbEGF-like7 domain was created based on the Protein data bank (PDB) template 1EMN (47% sequence identity), which demonstrated the solution structure of a pair of cbEGF-like domains of human fibrillin-1 [12]. PyMOL 1.1r1 was used to display the structure file and to predict the potential consequence of the mutation.

## Results

### Clinical features

**Figure 2:**
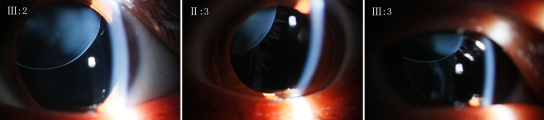
Slit lamp photographs of the right eye of the affected family members (III:2, II:3, and III:3 from left to right) after the pupils were dilated, showing ectopia lentis (superonasally).

All affected family members (II:3, III:2, and III:3) showed similar clinical symptoms: bilateral EL (Figure 2) was discovered in the three patients, and none of them displayed skeletal or cardiovascular abnormalities. The unaffected family members, including a spouse (II:5, III:1, and II:4), appeared normal.

### Mutation analysis

**Figure 3:**
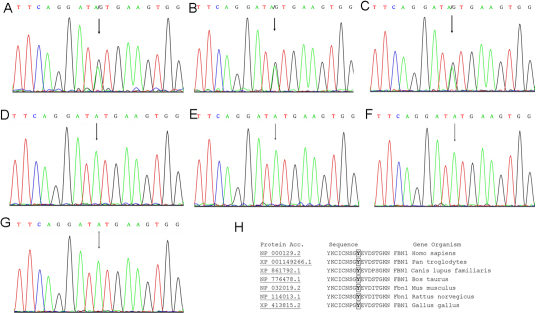
A *FBN1* mutation in exon 18. **A-C**: A heterozygous A>G transition (indicated by the arrow) resulted in the substitution of tyrosine by cysteine (Y754C) in the proband, patient II:3 and III:3 respectively. **D-F**: The corresponding normal sequence in the unaffected family member II:5, III:1, and spouse II:4, respectively. **G**: The corresponding normal sequence in a healthy control. **H**: The sequence alignment of FBN1 orthologs surrounding mutated site using ClustalW. The tyrosine^754^ of human FBN1 protein is highly conserved in several species. These sequences were selected from the NCBI database.

Direct sequencing of *FBN1* revealed a heterozygous mutation, c.2262A>G in exon 18, which resulted in the substitution of tyrosine by cysteine (p.Y754C; Figure 3A). The mutation identified in the proband was also found in II:3 and III:3 (Figure 3B,C). No mutation was detected in the healthy family members (II:5, III:1, and II:4; Figure 3D-F) or any of the 100 unrelated control subjects (Figure 3G).

### Potential consequences of the mutation

**Figure 4:**
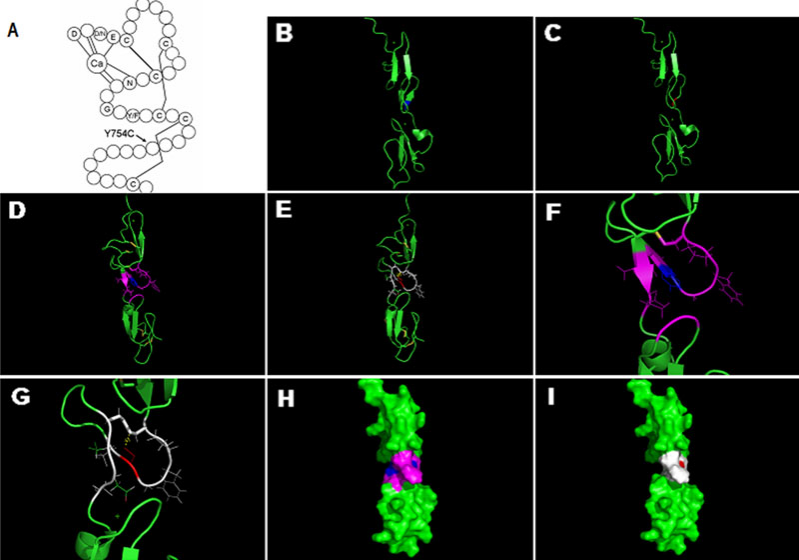
Structure analyses of the missense mutation in the calcium binding (cb) epidermal growth factor (EGF)-like7 domain. **A**: The consensus secondary structure of a prototypical cbEGF-like domain. Calcium binding in the NH_2_-terminal region of the wild-type domain is mediated by the consensus sequence (D/N) -X- (D/N) (E/Q) Xm (D/N) Xn (Y/F; m and n are variables), and highly conserved amino acids are identified by their single-letter amino acid code. The letter C in the schematic represents the highly conserved cysteine of cbEGF-like domain, and the lines between cysteine represent disulfide bridges. The mutation p.Y754C located at the region between the last two cysteines of the domain, which could probably interfere with the disulfide bond formation between the two cysteines. **B**: The 3D structure of the wild cbEGF-like7–8 domains, which are created based on the Protein Data Bank (PDB) template 1EMN (47% sequence identity) by PyMOL 1.1r1. The blue represents the unaffected tyrosine. **C**: The potential conformation change of the mutation. The red represents the substitute cysteine, where the double β-sheet transformed to a loop-region. **D** and **E**: The 3D structure of domains in **B** and **C**, respectively. The yellow lines represent disulfide bonds, and the blue represents the unaffected tyrosine. The purple displays the residues within the distance of 4Å with tyrosine^754^, and double β-sheet between obligatory glycine^753^ and aspartic^765^. The red represents the substitute cysteine, which absents the benzene ring. The yellow dashed line represents the potential disulfide bond formation between the introduced cysteine^754^ and cysteine^750^, which would probably disrupt the disulfide bond between cysteine^750^ and cysteine^763^. The white displays the residues within the distance of 4Å with cysteine^754^; compared with the purple wild type, β-turn, which forms the calcium binding pocket of cbEGF-like8, is farther in distance with cysteine^754^. **F** and **G**: A zoom-in change of **D** and **E**, respectively. **H** and **I**: The surface of the wild and mutant cbEGF-like7–8 domains, respectively. The colors correspond to that of figure **D** and **E**. The surface area of the mutant region is smaller than that of the wild. In summary, the conformation of the mutant domain is likely to be strongly altered, to include neighboring domain as well.

This missense mutation c.2262A>G resulted in the substitution of tyrosine by cysteine at codon 754 in the cbEGF-like7 domain. The tyrosine^754^ residue, which was present in 18/43 (42%) cbEGF-like modules in fibrillin-1, was localized in a β-sheet between obligatory cysteine residues at the C5 and C6 positions. Protein conservation analysis showed that the tyrosine^754^ was highly conserved among seven mammalian species (Figure 3H). Secondary structure analysis of the cbEGF-like7 domain revealed that the mutant residue was located at the region between cysteine^750^ and cysteine^763^ (Figure 4A). Prediction by PyMOL 1.1r1 showed a loss of benzene ring due to the Y754C mutation and an additional disulfide bond formation between cysteine^750^ and cysteine^754^, which simultaneously disrupted the conserved disulfide bond between cysteine^750^ and cysteine^763^. These led to a critical structure change: the β-sheet between obligatory glycine^753^ and aspartic^765^ transformed to a loop-region. domain change of this nature could probably alter the volume of the calcium binding pocket of the posterior cbEGF-like domain because the distance between cysteine^754^ and the residues that formed β-turn of the pocket become farther than in the wild type (Figure 4B −4G). In addition, the protein surface area of the mutant region was smaller than that of the wild type (Figure 4H,I). In summary, the conformation and function of the mutant domain were likely to be strongly altered by the presence of this mutation, extending to neighboring domains as well.

## Discussion

In this study, we identified a heterozygous *FBN1* mutation (c.2262A>G) in a Northeast Chinese family affected with EL. This missense mutation introduced an additional cysteine residue by substitution of a highly conserved tyrosine residue within the cbEGF-like7 module.

This mutation, p.Y754C, has been previously reported in three other families: a large family of European and Australian Aboriginal origin, and two Central Chinese families [16-18]. All three families met the criteria for a diagnosis of MFS and, interestingly, the overwhelming majority of members from each family had lens subluxation, with or without cardiovascular and skeletal abnormalities. In our study, the only manifestation in the family was lens subluxation. These data demonstrate a complete correlation between p.Y754C mutation and lens subluxation. Nevertheless, more studies should be done to confirm this conclusion. To date, eight novel mutations have been published in the UMD database in the cbEGF-like7 domain, and seven of them are mutations creating or substituting cysteine residues [19-23]. A striking result of a recent study involving over a thousand probands with MFS and *FBN1* mutations is the strong correlation found between EL and the presence of a mutation affecting a cysteine residue [14], which also confirms earlier conclusions on a smaller sample [16,24-27]. These findings suggest that cysteine residues may have a critical function in suspensory ligaments of the eyes and led to the speculation that the pathophysiology of ectopia lentis is related to a disruption of the structural function of fibrillin-1 in the 10–12 nm extracellular microfibrils in the ciliary zonule [14,27].

It is clear that EGF-like domains play a major role in the pathogenesis of fibrillinopathies [28]. Each cbEGF-like domain of fibrillin-1 contains six highly conserved cysteine residues that form three intra-domain disulphide bonds generating an anti-parallel β-pleated sheet conformation and a consensus sequence for calcium binding in the NH_2_-terminal region [12,29]. In the cbEGF-like7 domain, the intra-domain disulphide bonds are formed between cysteine^727^ and cysteine^739^, cysteine^734^ and cysteine^748^, and cysteine ^750^ and cysteine ^763^, respectively, according to the UMD database. The mutation found in our study, located at the region between cysteine ^750^ and cysteine ^763^, introduced an extra cysteine. A detailed study performed by PyMOl showed that the extra cysteine could probably disrupt the third conserved disulfide bond and introduce an additional one, which would break the β-sheet between obligatory cysteine ^750^ and aspartic^765^. It is evident that three disulfide bonds are required to maintain the cbEGF-like module-fold. The loss or addition of cysteine residues would result in module misfolding, which in turn may have deleterious effects on the global structure of fibrillin-1 and delay intracellular processing and/or secretion from the cell that lead to severe reduction of matrix deposition [30-32]. Prediction by PyMOL also indicated that such a change of the domain could probably influence the packing interaction of the cbEGF-like7 and cbEGF-like8 domain and alter the volume of the calcium-binding pocket of the adjacent cbEGF-like8 domain, which may affect calcium binding affinity of the cbEGF-like8 and expose the loop between the cysteines at the C3 and C4 positions to proteases. This would cause unexpected endoplasmic reticulum retention of the protein, consistent with a protein folding defect, and increase the susceptibility of fibrillin peptides to proteolysis [15]. In support of our speculation, a recent study by Vollbrandt et al. [33] demonstrated that a C750G substitution of *FBN1* that disrupt the cysteine^750^-cysteine^763^ disulfide bond of cbEGF-like7 caused increased proteolytic susceptibility of cbEGF-like8. On the protein level, the misfolded domain of the mutant protein may be degraded by intracellular mechanisms or retained within the cell, or escape from quality control surveillance in the cell. For the latter, on encountering the extracellular space, mutant proteins may be rapidly degraded by proteases in the surrounding environment or may subsequently disrupt a specific protein–protein interaction required for the assembly of fibrillin-1 or interactions of microfibrils with other cell-matrix components [34]. This theory can also be proved by immunohistochemical staining of fibrillin in fibroblast cultures of patients from the Australia family carrying the same p.Y754C mutation: in normal fibroblasts, most of the fibrillin was located in the extracellular area, and the fibers were long, smooth, and fine in appearance; in mutant fibroblasts, most of the fibrillin was found within the cells, and the limited amount of fibrillin in the extracellular matrix was disorganized and appeared clumped rather than fibrous [16].

Collectively, evidences from our study and published data supported that the p.Y754C mutation was the causative mutation for EL in this family. Mutations involving cysteine substitutions in cbEGF-like domains of FBN1 play a critical role in the pathogenesis of EL. Our study offers the first predictions on the structural and functional consequences of this mutation in detail. The result expands the genotype-phenotype spectrum of *FBN1* and helps the study of the molecular pathogenesis of EL and Marfan-related disorders.

## Appendix 1. Primers used for *FBN1 *amplification.

Summary of the primers used for the amplification of *FBN1 *exons. Sequences are given in the 5'→3' direction. To access the data, click or select the words “Appendix 1.” This will initiate the download of a compressed (pdf) archive that contains the file.
